# Use of Different Nutrients to Improve the Fermentation Performances of *Lactiplantibacillus pentosus* OM13 during the Production of Sevillian Style Green Table Olives

**DOI:** 10.3390/microorganisms11040825

**Published:** 2023-03-23

**Authors:** Antonio Alfonzo, Vincenzo Naselli, Raimondo Gaglio, Luca Settanni, Onofrio Corona, Francesco La Croce, Paola Vagnoli, Sibylle Krieger-Weber, Nicola Francesca, Giancarlo Moschetti

**Affiliations:** 1 Department of Agricultural, Food and Forest Sciences (SAAF), University of Palermo, Viale delle Scienze Bldg. 5, Ent. C, 90128 Palermo, Italy; 2 Geolive Belice S.r.l., S.S. 115 Km dir. Marinella, 91022 Castelvetrano, Italy; 3 Lallemand Italia, Via Rossini 14/B, 37060 Castel D’Azzano, Italy; 4 Lallemand, Office Korntal-Münchingen, In den Seiten 53, 70825 Korntal-Münchingen, Germany

**Keywords:** table olives, start culture, lactic acid bacteria, nutrient, volatile organic compounds

## Abstract

The aim of this study was to evaluate the fermentation performance of the commercial starter *Lactiplantibacillus pentosus* OM13 with four nutrients (A, B, C, and D) that differed in the following ingredients: starch, sugars, maltodextrin, inactivated yeast, inactivated yeast rich in amino acids, inactivated yeast rich in mannoproteins, and salt (NaCl). For this purpose, six different experimental productions of Nocellara del Belice table olives were carried out. During transformation, the fermentation process was monitored by measuring pH and plate counts for lactic acid bacteria (LAB), yeasts, Enterobacteriaceae, Staphylococcaceae, and Pseudodomondaceae populations. At the end of the production process, each trial was subjected to volatile organic compound analysis and sensory evaluation. The addition of the different nutrients resulted in a significant reduction in pH (around 2.5 points) after 3 days of fermentation. At the same time, a significant increase in the number of LAB populations (> 6.6 log CFU/mL) was observed for all trials. Volatile organic compound (VOC) analysis revealed the presence of 39 compounds. In this study, nutrient C was optimal for improving the fermentation activity of *L. pentosus* OM13. These results provide elements for the implementation of experimental protocols to reduce product losses and improve sensory characteristics.

## 1. Introduction

Table olives have been produced through spontaneous fermentation for decades, but this process proved to be unpredictable and uncontrollable. The lack of standardisation of the table olive fermentation process can cause issues in terms of the sensory profile and safety of the final products [1]. Hence, table olives, especially at industrial level, are commonly processed with the use of selected starter cultures, which are able to warrant high sensory quality and safety at the end of transformation [2].

The biological debittering of drupes during natural fermentations relies on the enzymatic activity of several lactic acid bacteria (LAB), mainly *Lactiplantibacillus plantarum* and *Lactiplantibacillus pentosus* [1,3,4]. Moreover, LAB acidify the product by the generation of lactic acid, which exerts an inhibitory effect on the undesirable microorganisms [5]. In the last decade, research activities have been specifically carried out to select starter LAB to produce fermented green table olives, with the main objective being to improve product quality [6]. LAB can control and reduce fermentation times; they improve product stability, extend shelf-life, and ameliorate the sensory profile, providing key factors for the industrial production of table olives [7]. To this purpose, LAB species most commonly used as starter cultures are *L. plantarum, L. pentosus, Lacticaseibacillus paracasei,* and *Lacticaseibacillus rhamnosus* [8,9,10,11].

A primary aspect of industrial table olive production is the rapid acidification because values below 4.5 create a hostile environment for the development of undesirable microorganisms. In the production of Seville-style fermented olives, a limiting factor in the early stages of fermentation is the suboptimal pH for the LAB starters. This is compounded by nutritional and environmental conditions that limit LAB growth [12,13,14]. Some authors have proposed to solve the problem of brine acidification by using lactic acid during the early stages of fermentation [15]. The addition of nutrients (reducing sugars, vitamins, and amino acids) allowed certain strains of *L. plantarum*, isolated from table olive fermentation brines, to improve the rate of acidification and the amount of lactic acid produced [16]. The addition of certain nutrients to the brine, concomitantly to starter strain inoculation, can increase fermentation efficiency [17]. Particularly, green table olives inoculated with *L. pentosus* showed a lower cell density of Enterobacteriaceae and Pseudomonadaceae when glucose was added together with the starter strain [17]. Further studies have confirmed that the addition of a specific nutrient can improve the fermentation performance of *L. pentosus* in table olives produced in the Spanish-style [9,18]. The addition of nutrients is particularly important for the management of controlled fermentations of green table olives, but their use has not been optimized yet.

Based on the previous studies, the main aim of the current study was to evaluate the behaviour of *L. pentosus* OM13, a commercial starter industrially used for the production of table olives [9], in the presence of different nutrients (varying for the amount of sugars, presence/absence of starch, maltodextrine, salt, and *Saccharomyces cerevisiae* inactivated yeast) in order to improve the fermentation phase of Nocellara del Belice cultivar green table olives. Microbiological, physicochemical, and sensory parameters were carried out to assess the variations among the trials.

## 2. Materials and Methods

### 2.1. Experimental Design, Table Olive Manufacturing and Sample Collection

**Figure 1:**
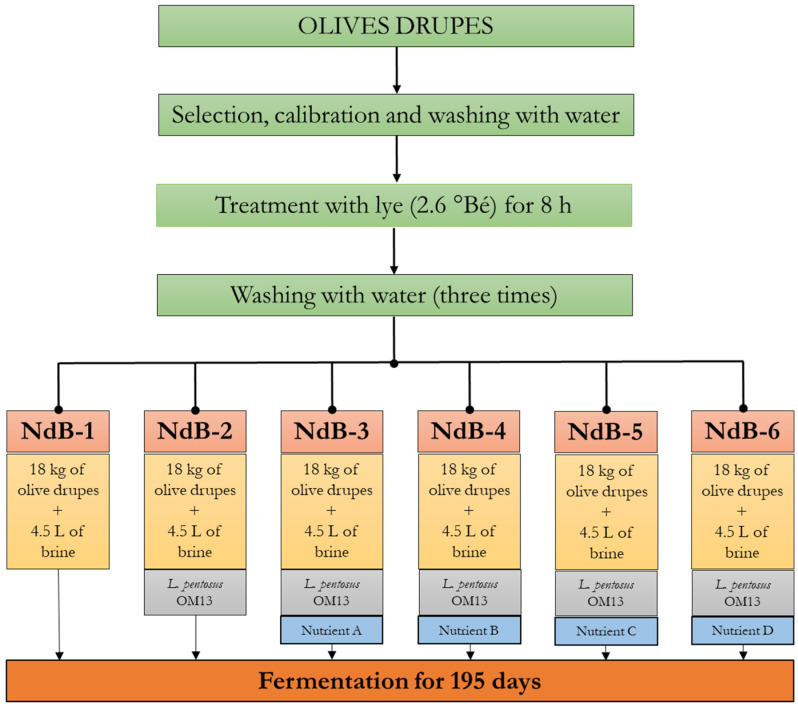
Experimental design for green table olive manufacturing. Abbreviation: °Bé, Baumè degree; NdB-1 and NdB-2 codes refer to the control trials; NdB-3, NdB-4, NdB-5, and NdB-6 codes refer to the experimental trials; *L.*, *Lactiplantibacillus*.

The Nocellara del Belice drupes were harvested when ripe. The fruits were calibrated, rinsed with water and processed according to the Sevillian method: the olives were treated with lye (2.6 °Bé) for 8 h to minimise the bitterness and then washed three times in succession to remove the lye residues. According to the experimental design, 18 kg of olive drupes were transferred into 25 L volume plastic containers and added with 4.5 L of brine (10% *w*/*v* NaCl). The experimental design included six trials (NdB-1–NdB-6; Figure 1). Except for NdB-1, which followed a spontaneous fermentation, all experimental productions were inoculated (0.0083 g/Kg of olive) with Lal’Olive Crispy *L. pentosus* OM13 (Lallemand Inc., Montreal, Canada) in freeze-dried form, containing approximately 1.1 × 10^9^ colony forming units (CFU)/g and food grade maltodextrine as the carrier. NdB-2 represented the control trial. Strain performances were assessed with four different nutrients (Table 1), all added at 1 g/kg of olives: Nutrient A–D were added to Trial NdB-3–Ndb-6, respectively. The four nutrients were supplied by Lallemand Inc. (Montreal, Canada). All productions were fermented at room temperature (average value of 19 ± 3 °C) for 195 days and were periodically monitored. There were two separate productions (experimental replicates) performed over two weeks in October and November 2021 at the Geolive Belice S.r.l. company located in Castelvetrano (Trapani region, Sicily, Italy) (37° 38′ 16″ N/12° 49′ 55″ E).

Brine samples were collected immediately after starter culture inoculation and nutrient addition (day 0), as well as after 3, 6, 9, 15, 35, 65, 143, and 195 days of fermentation. Brines (100 mL/sample/sampling phase) were placed into sterile containers, transported at 4 °C to the Agricultural Microbiology laboratories, University of Palermo, and subjected to the microbiological analysis. Olive table productions were carried out in triplicate (three vats per trial—technical repeats).

### 2.2. Physico-Chemical and Microbiological Analysis

The pH of the brine was determined by the direct immersion of a pH meter from Hanna Instruments HI98165 (Ronchi di Villafranca Padovana, Italy). All measurements were carried out in triplicate.

Brine samples were serially diluted in Ringer’s solution (Sigma-Aldrich, Milan, Italy). The microbial populations investigated were as follows: rod LAB on de Man–Rogosa–Sharpe (MRS) agar added with cycloeximide (10 mg/mL) that were anaerobically incubated for 48 h at 30 °C; yeasts on Dichloran Rose–Bengal Chloramphenicol agar (DRBC) that were aerobically incubated for 120 h at 25 °C; Enterobacteriaceae on Violet Red Bile Glucose Agar (VRBGA) that were aerobically incubated for 24 h at 37 °C; staphylococci and coagulase-positive staphylococci (CPS) on Baird-Parker (BP), added with a RPF supplement, that were aerobically incubated for 48 h at 37 °C; *Pseudomonas* spp. on Pseudomonas agar with CFC supplement (PCFC) that were aerobically incubated for 48 h at 30 °C [19]. Cells were inoculated by the spread plate technique on DRBC, BP, and PCFC, while the pour plate technique was applied for analysis on MRS and VRBGA. All growth media were purchased from Oxoid (Milan, Italy). All analyses were performed in triplicate, and the results expressed as the average number of colony-forming units (CFU)/mL.

### 2.3. Dominance of Lactiplantibacillus pentosus Isolates

After growth on MRS agar, LAB isolates were collected. For each morphology, at least five colonies were collected from the agar plates and, subsequently, purified in the same growth medium. Cell morphology was determined by microscopy. For each sample, at least five morphologically identical isolates were maintained and stored at −80 °C. Bacteria were distinguished as Gram+/Gram− by the Gregersen KOH method [20], and then, they were assessed for the presence of the enzyme catalase (5% *w*/*v*, H_2_O_2_). Phenotypic grouping was performed as described by Martorana et al. [21].

Presumptive LAB isolates were subjected to genotypic investigation after DNA from extraction occurred, according to Ruzauskas et al. [22]. During fermentation, the dominance of strain OM13 was measured by random amplification polymorphic DNA-PCR (RAPD-PCR) in a 25 L reaction mix using primer M13 [23]. All amplifications were performed using Swift max PRO (Esco Healthcare, Singapore); the electrophoresis runs were carried out on 2% agarose gel (*w*/*v*) in 1× TBE buffer, applying 100 V for 2 h and 20 min. SYBR^®^ Safe DNA gel stain (Invitrogen, Life Technologies, Monza, Italy) was added directly to agarose gel (1 μL/10 mL of agarose gel) before solidification. The bands were visualized by a UV transilluminator and captured by Gel Doc 1000 Video Gel Documentation System (BioRad, Richmond, VI, USA). Band height was compared to GeneRuler marker 100 bp DNA Ladder Plus (Thermo Fisher Scientific, Waltham, MA, USA). RAPD patterns were analysed with the Gelcompar II software, version 6.5 (Applied-Maths, Sint-Martens-Latem, Belgium). Using 16S rRNA gene sequencing, 10% of the LAB isolates in each group, with the same polymorphic profile, were identified to species level [24]. DNA sequencing reactions were performed at AGRIVET laboratory (Palermo, Italy). Sequence identity was determined by searching BlastN against the NCBI non-redundant sequence database (http://www.ncbi.nlm.nih.gov, accessed on 13 March 2023) and EZTaxon (http://www.ezbiocloud.net accessed on 13 March 2023).

LAB strains belonging to the *L. plantarum* group were confirmed by multiplex PCR of the recA gene with species-specific primers for *L. pentosus*/*plantarum*/*paraplantarum* [25].

### 2.4. Volatile Organic Compound Identification

After 195 days of fermentation, VOCs were detected utilising the Solid Phase Micro-Extraction approach in Head Space, followed by Gas Chromatography/Mass Spectrometry (HS-SPMEGC/MS) [26]. After homogenising 5 g of drupes, an aliquot of 0.5 g was transferred into 2 mL vials containing pierceable silicone rubber septa covered with polytetrafluoroethylene (PTFE) film. The internal standard was 50 microliters of 2-pentanol-4-methyl methanol solution (0.981 mg/mL). The SUPELCO SPME (Bellefonte, PA, USA) fibre holder and utilised fibre were both coated with divinylbenzene/carboxen/polydimethylsiloxane. To reach equilibrium, vials were heated at a controlled temperature (40 ± 0.5 °C) for 30 min. The GC-MS setting followed the procedure provided by Corona [27]. Volatile organic compounds were identified through a comparison of the mass spectra and GC retention times with those of the pure commercial standard compounds, as well as by comparing their mass spectra with those within the NIST/EPA/NIH Mass Spectral Library database (Version 2.0d, build 2005, National Institute of Standards and Technology, Gaithersburg, MD, USA). For volatile organic compounds without the commercially available standard, their identification was conducted by matching their mass spectrum with those of the NIST library or those reported in the literature. The quantification of the compounds identified was expressed in relation to the internal standard used. All analyses were carried out in triplicate.

### 2.5. Sensory Evaluation

At the end of fermentation (day 195), the descriptive technique ISO 13299:2016 [28], as previously published [21,29,30,31], was used to evaluate the sensory profiles of the olives. In preliminary sessions, 14 judges (7 female and 7 male, aged 21 to 58 years) were trained using different samples of commercial table olives of the Nocellara del Belice variety in order to establish a common vocabulary for describing the sensory attributes of the experimental samples and to familiarise them with the scales and procedures. In order to eliminate any ambiguity regarding the appropriate interpretation, each characteristic was thoroughly clarified and detailed.

The sample was evaluated using the sensory attributes used by the panellists with a frequency > 60%. Each experimental product was evaluated with 13 descriptors considering visual characteristics (brightness and intensity of green colour), odour (green olive aroma), rheological characteristics (crispness), and taste (juicy, acid, bitter, salty, astringent, sweet, and overall acceptability). The possible presence of abnormal fermentation or other defects characterising the olives were defined by attributes such as off-odour and off-flavour [32]. The evaluation of the olive samples took place in individual booths illuminated by white incandescent light, and the hedonic scale used ranged from 1.00 (no sensation) to 9 (extremely intense sensation).

### 2.6. Statistical Analysis

ANOVA procedure was used to statistically analyse data from microbiological analysis, acidification dynamics (pH), concentration of VOCs, and sensory scores. Tukey’s post-hoc procedure was used for pairwise comparisons. Statistical significance was assigned to *p* ≤ 0.001. According to Gaglio et al. [33], heat map cluster analysis (HMCA) was used to identify the distribution of VOCs in the treatments. Agglomerative Hierarchical Clustering analysis (AHC) was used to separate the experimental products based on their dissimilarity (expressed in Euclidean distances and using Ward’s method) among the trials based on the sensory attribute scores. All analyses were carried out using the XLStat software version 2019.2.2. (Addinsoft, New York, NY, USA).

## 3. Results

### 3.1. Brine Acidification

The dynamics of the pH of brines, during the 195 days of table olive production, are depicted in Table 2.

Until day 6, *L. pentosus* OM13-inoculated trials were characterized by lower pH values than control treatment (NdB-1). NdB-4–NdB-6 trials showed significantly lower pH values on day 9 (from 4.96 to 5.04) as compared to NdB-3 (5.44), NdB-2 (5.64), and NdB-1(6.68). On day 15, the pH of all inoculated trials ranged between 4.65 (NdB-5) and 5.11 (NdB-2), whereas the pH of the spontaneously fermenting trials (NdB-1) exceeded 6. The pH of all experiments gradually decreased over the next few days. The lowest pH level at the end of the monitoring period (195 days) was 4.35, and it was displayed by NdB-5 brine.

The nutrient supplied clearly influenced pH changes. In particular, all nutrients determined a consistent and quick pH lowering. Nutrient A showed lower pH values up to the 9th day of fermentation compared to the trials without nutrients. From day 15 to the end of the process, no differences were observed between the NdB-2 and NdB-3 treatments.

### 3.2. Dynamics of Microbial Populations

#### 3.2.1. Lactic Acid Bacteria

**Figure 2:**
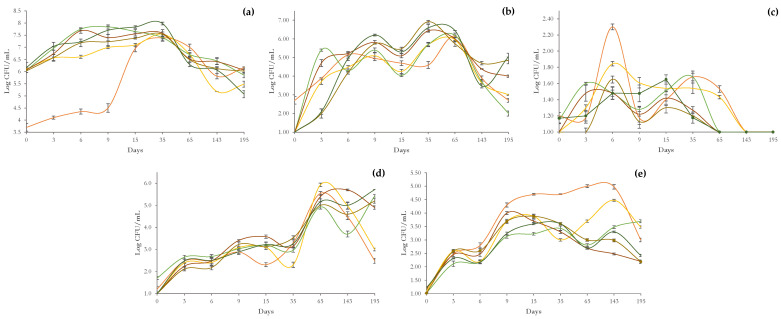
Trend of microbial populations determined during 195 days of fermentation: (**a**) lactic acid bacteria; (**b**) yeasts; (**c**) Enterobacteriaceae; (**d**) Pseudomonadaceae; (**e**) Staphylococcaceae. Colours: ▬, NdB-1 (spontaneous fermentation); ▬, NdB-2 (*L. pentosus* OM13); ▬, NdB-3 (*L. pentosus* OM13 + nutrient A); ▬, NdB-4 (*L. pentosus* OM13 + nutrient B); ▬, NdB-5 (*L. pentosus* OM13 + nutrient C); ▬, NdB-6 (*L. pentosus* 255 OM13 + nutrient D).

Figure 2a shows data on dynamics of LAB populations. Immediately after inoculation of the starter strain *L. pentosus* OM13, the experimental productions revealed count levels above 6.0 Log CFU/mL, while in the control (NdB-1) trial, the levels were slightly above 4 log cycles. Until day 15, trial NdB-1 displayed the lowest LAB cell density (6.8 Log CFU/mL), whereas the inoculated trials showed a constant increase in LAB count values to levels around 7.0 Log CFU/mL. Trial NdB-3 exhibited the greatest LAB load (7.8 Log CFU/mL) until day 9. Only at day 35, LAB counts of trial NdB-1 (7.4 Log CFU/mL) were comparable to those observed in the inoculated trials (7.4–8 Log CFU/mL). At day 65, LAB populations in the control trial (NdB-1) were 7.0 CFU/mL, but they were counted at lower levels (6.29–6.70 CFU/mL) in the inoculated trials (NdB-2 to NdB-6). After 143 days, the trials NdB-1 and NdB-2 were characterized by LAB levels lower than 6 log cycles. The differences in counts of LAB populations detected at the conclusion of the monitoring period (195 days) were variable and within the range of 5.1–6.2 Log CFU/mL independently of the presence/absence and type of nutrient.

#### 3.2.2. Yeasts

The trend of the yeast populations during table olive production is depicted in Figure 2b. At time zero, only the spontaneously fermented trial NdB-1 showed the presence of yeasts (2.7 Log CFU/mL) above the detection limit (1.0 Log CFU/mL). During the 195 days of monitoring, the count levels fluctuated in the range of 1.0–6.9 Log CFU/mL. Overall, the trials NdB-5 and NdB-6 showed the highest microbial count levels after 15 days (5.5 and 5.3 Log CFU/mL, respectively) and at 195 days (5 and 4.9 Log CFU/mL, respectively).

#### 3.2.3. Enterobacteriaceae

Across the monitoring phase (195 days), members of the Enterobacteriaceae family were between 1.0 and 2.3 Log CFU/mL (Figure 2c).

On day 6 of fermentation, the highest value was observed in the trial NdB-1. At day 65, with the exception of the trials with no nutrient addition (NdB-1 and NdB-2), the microbial count values were below the detection limits. From day 143 until the end of the process, the presence of Enterobacteriaceae was not detected in any experimental production.

#### 3.2.4. Pseudomonadaceae

The Pseudomonadaceae group increased constantly until day 35, with count values ranging from 1.0 to 3.6 CFU/mL. (Figure 2d).

At day 65, all trials had an increase in microbial count values of about 2 log cycles. The nutrient-free trials (NdB-1 and NdB-2) showed the lowest levels at the end of the monitoring (2.5 Log CFU/mL and 3.0 Log CFU/mL, respectively).

#### 3.2.5. Staphylococcaceae

The Staphylococcaceae trend across the process fluctuated between 1.0–5.0 Log CFU/mL (Figure 2e).

After the first 15 days, the lowest values were detected in NdB-3 (3.23 Log CFU/mL). NdB-1 achieved the highest value (5.0 Log CFU/mL) at day 65. From day 65 to day 195, the lowest concentrations (2.7 to 2.2 Log CFU/mL) were observed for trial NdB-4. The trials Ndb-4, NdB-5, and NdB-6, at 195 days, reached count levels of less than 3 log cycles. Coagulase-positive staphylococci were not detected in any samples.

### 3.3. Genotypic Characterisation of Lactic Acid Bacteria

**Figure 3:**
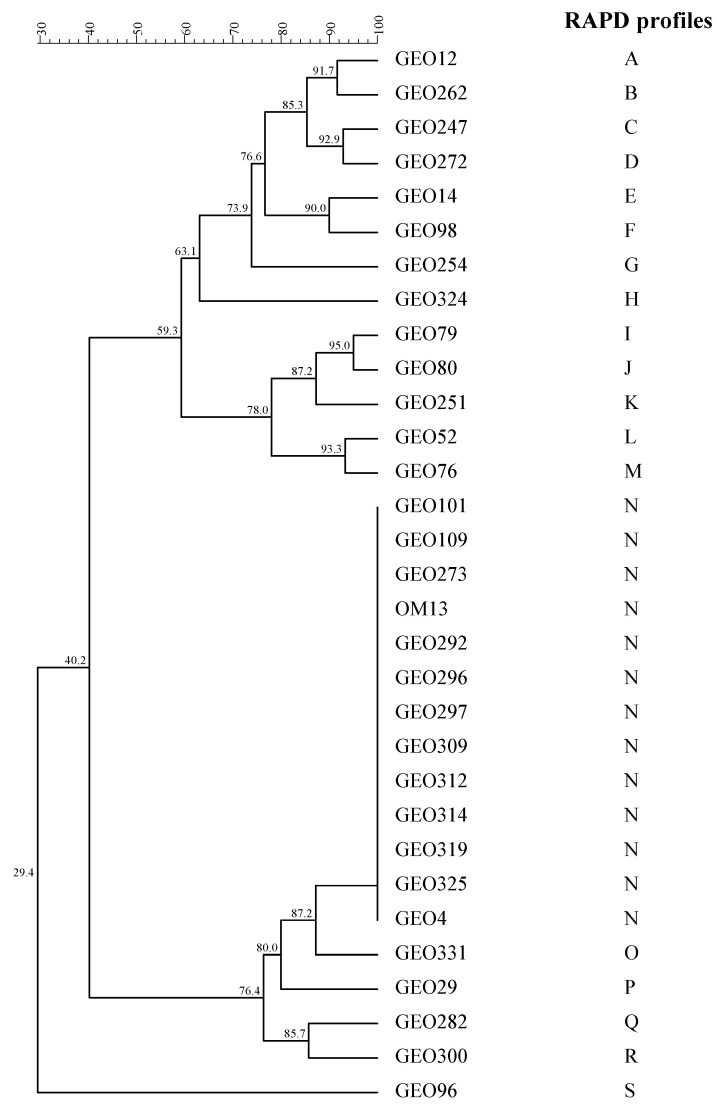
Dendrogram obtained from RAPD-PCR patterns of *Lactiplantibacillus pentosus* strains isolated from Nocellara del Belice table olive samples during fermentation.

Significant numbers of colonies were collected from the highest dilutions of the cell suspensions of all samples. The majority of isolates showed the typical rod-shaped cell morphology of lactobacilli. Following a genotypic investigation (16S rRNA gene sequencing and multiplex PCR of the *recA* gene), 106 isolates were identified as *L. pentosus species* and subjected to RAPD-PCR analysis. A direct comparison of RAPD profiles revealed that the most frequently isolated strain in the inoculated trials was *L. pentosus* OM13. The polymorphic profiles of 83% of the isolates revealed a high similarity (>95%), with the reference starter *L. pentosus* OM13 strain utilized as inoculum. Figure 3 shows the dendrogram with 19 different RAPD profiles. The most abundant RAPD profile (N) was represented by 88 isolates (only 12 isolates are shown) with a polymorphic profile identical to the *L. pentosus* OM13. The other RAPD profiles (No. 18) were associated with LAB strains, but they were different from the starter strain OM13. The spontaneous fermentation (NdB-1) trial did not host any strain with a polymorphic profile similar to the starter strain *L. pentosus* OM13. However, the dominant LAB species were *L. plantarum* (Acc. No. OQ596979) and *L. pentosus* (Acc. No. OQ596980).

### 3.4. Volatile Organic Compound Composition

VOCs generated by table olives at the end of production (day 195) are shown in Table 3. There were 39 compounds belonging to the following classes identified: acids (*n* = 5), alcohols (*n* = 8), aldehydes (*n* = 7), aromatic hydrocarbons (*n* = 2), esters (*n* = 12), ketones (*n*= 1), and phenols (*n*= 4). In terms of quantity, phenolic compounds were the most prevalent class, followed by those of acids and esters. Among phenols, creosol was detected at the highest level in all experimental production, and the highest concentration was registered for the trial NdB-6 (6176.69 μg/kg). The main acid was acetic acid, which was present at very high concentrations in Ndb-6 (3190.57 μg/kg). Pentanoic acid was only detected in Ndb-2.

Among esters, cis-3-hexenylacetate, octyl acetate, and butyrolactone were identified in all experimental productions. The ester present at the highest amount was ethyl cyclohexanecarboxylate (3036.13 μg/kg) in NdB-2. Among the other classes of VOCs, phenylethyl alcohol (1189.57 μg/kg in NdB-6), Benzaldehyde (2200.07 μg/kg in NdB-5), and alpha-cubebene (125.17 μg/kg in NdB-1) were the compounds detected at consistent levels. Altogether, the highest number of VOCs was identified in NdB-2 (*n* = 35), followed by trials NdB-4 and NdB-6 (*n* = 28), NdB-1 and NdB-5 (*n* = 26), and finally, NdB-3 (*n* = 25). For all the compounds detected, the trials where nutrient C (NdB-5) was added showed the highest concentrations for 12 VOCs (3-methyl- 1-butanol, benzaldehyde, benzaldehyde-2-5-dimethyl, isophthaldehyde, octanal, styrene, butyrolactone, ethyl dihydrocinnamate, octyl acetate, phenylmethyl acetate, 4-Ethylacetophenone, and phenol).

**Figure 4:**
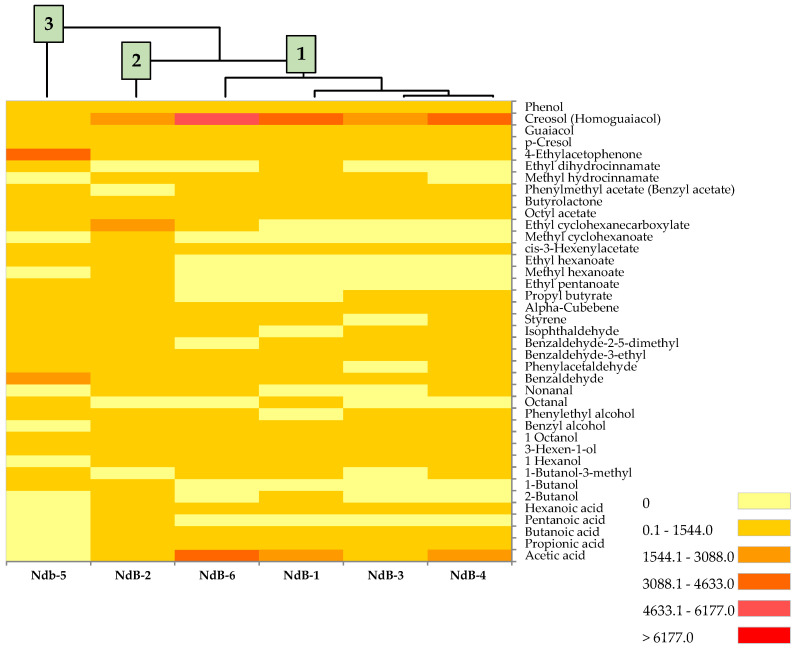
Distribution of volatile organic compounds within table olive experimental production. The relative concentration of each VOC is shown in the heat map plot for each trial. The hierarchical dendrogram is based on the values of VOCs. Abbreviations: NdB-1, spontaneous fermentation; NdB-2, *L. pentosus* OM13; NdB-3, *L. pentosus* OM13 + nutrient A; NdB-4, *L. pentosus* OM13 + nutrient B; NdB-5, *L. pentosus* OM13 + nutrient C; NdB-6, *L. pentosus* OM13 + nutrient D.

Figure 4 shows the cluster analysis dendrogram and heat map based on the VOC levels and distribution in the six experimental products. The relationships between table olive productions depend on the concentrations of the different VOCs. The cluster analysis revealed the existence of three main clusters. Cluster 1 consisted of the trials NdB-1, NdB-3, NdB-4, and NdB-5. Clusters 2 and 3 were represented by trials NdB-2 and NdB-5, respectively. The creosol content affected the differentiation of cluster 1. Cluster 2 was differentiated by ethyl cyclohexanecarboxylate and cluster 3 was differentiated by 4-ethylacetophenone.

### 3.5. Sensory Characterisation of Tables Olives

The different trials were sensory analysed at the conclusion of the transformation process (195 days), and the results are reported in Table 4.

Among the 13 attributes examined, 9 revealed statistically significant differences. Trial NdB-5 received the highest scores for crispness (7.00), green olive aroma (6.13), juicy (6.18), salt (6.13), sweet (4.32), and overall acceptability (7.80). Spontaneously fermented production (NdB-1) showed the highest values for off-flavours (4.82), off-odours (3.27), and salt (3.27). In terms of overall acceptability, the highest score was observed for trial NdB-5 (7.80), while the lowest was for trial NdB-1 (3.55).

**Figure 5:**
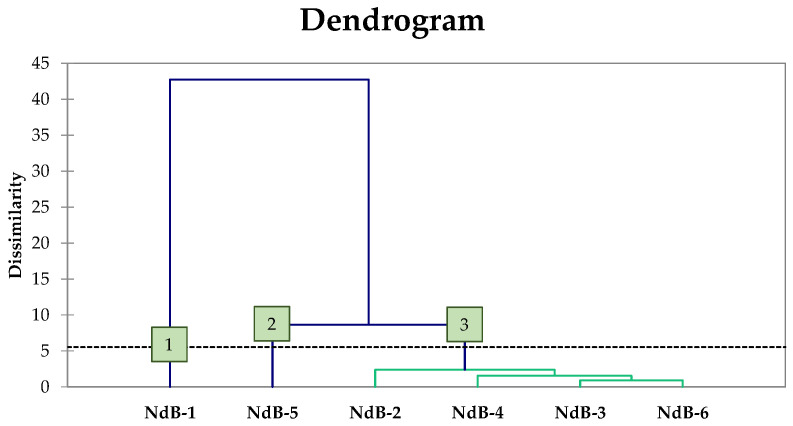
Dendrogram derived from the AHC of table olives based on the values of the attributes assessed during sensory analysis. Abbreviations: NdB-1, spontaneous fermentation; NdB-2, *L. pentosus* OM13; NdB-3, *L. pentosus* OM13 + nutrient A; NdB-4, *L. pentosus* OM13 + nutrient B; NdB-5, *L. pentosus* OM13 + nutrient C; NdB-6, *L. pentosus* OM13 + nutrient D.

AHC categorised the six experimental productions based on their mutual dissimilarity and relationship, considering all sensory traits together (Figure 5).

This analysis classified the trials using thirteen variables chosen based on sensory analysis results. With a dissimilarity of 5%, all experimental table olive products were clearly separated into three clusters. Cluster 3 was the most numerous, including four trials (NdB-2, NdB-3, NdB-4, and NdB-6). NdB-1 and NdB-5 represent Clusters 1 and 2, respectively. The parameters that had the greatest effect on trial cluster formation were juiciness and overall acceptability.

## 4. Discussion

In this study, four different nutrients were evaluated in order to improve the fermentative activity of *L. pentosus* OM13 in terms of brine acidification kinetics. The formulation of the four nutrients considers that the olives to be fermented represent a matrix low in sugars, so the addition of glucose promotes the growth of LAB starters. The amount of fermentable sugars varies greatly depending on the cultivar, climate, agronomic techniques, and harvest period [34]. Sugar was an indispensable component of all nutrients used to compensate for nutritional deficiencies. The maltodextrins were added in nutrients B, C, and D because of their known prebiotic activity on *Lactobacillus* spp. [35]. In addition, the presence of inactivated yeasts, not enriched and enriched in amino acids and mannoproteins, are known growth promoters of malolactic LAB, and they also improve the survival of probiotics [36,37,38]. In nutrient A, unlike the others, starch was added to replace inactivated yeasts, as some LAB are able to use this polysaccharide for their own growth [39].

A recently developed approach in this field is the use of nutrients as a support to increase the fermentative ability of starter LAB used in table olive production. In this study, the use of four different nutrients allowed a rapid colonisation of the starter culture and a rapid decrease in pH values. This result is in contrast with those reported by Martorana et al. [9] and Alfonzo et al. [18], where the production protocols did not include the addition of lactic acid before fermentation. The rapid reduction in pH during the first few days of fermentation is critical in preventing the growth of spoilage microorganisms, which might lead to the “gas pocket” or softening of drupes [40]. Trials involving nutrient addition (NdB-3, NdB-4, Ndb-5, and NdB-6) revealed the highest levels of LAB densities and, as a result, a rapid decrease in pH, in contrast to the NdB-1 control, where the lowest levels of LAB populations observed caused a slow process of brine acidification, as reported by Romeo [14]. In this context, nutrient utilization has been shown to significantly improve the dynamics of LAB microbial populations. The levels of LAB populations observed in the six treatments can be attributed to the different production protocols applied. Nutrient A positively influenced the LAB growth dynamics until the 9th day of fermentation, while a slow LAB growth dynamic was observed in the treatment without nutrient addition. The trend of LAB populations, found in spontaneously fermented production, showed the same trend, as described by Martorana et al. [9]. The ability of *L. pentosus* OM13 to dominate the indigenous LAB population was observed in both inoculated (NdB2 to NdB-6) and nutrient-supplemented (NdB-3 to NdB-6) experimental trials. The dominance of the starter culture is important in the guided fermentation of table olives because it ensures that the lactic fermentation was performed by the inoculated starter strain; furthermore, the absence of RAPD profiles in the control trial (NdB-1) allowed us to rule out any contamination of the starter strain used [1]. In accordance with what was reported by Bautista-Gallego et al. [41], the presence of yeasts in lye-treated olives was relatively low at the beginning of the fermentation process, but the trend observed is comparable to that reported by Alfonzo et al. [18]. In NdB-4, NdB-5, and in NdB-6, the presence of higher yeast microbial densities, from day 9 to day 65 of the process, was attributable to the presence of the sugars, maltodextrine, and inactivated yeasts present in nutrients B, C, and D. On the contrary, this composition did not affect the growth levels of the populations of the alterative and/or potentially pathogenic microorganisms monitored (Enterobacteriaceae, Pseudomonadaceae, and Staphylococcaceae), which showed a similar trend to that reported by Alfonzo et al. [18].

The experimental products were separated into three different groups using heat maps based on the VOC concentrations determined for each trial. The compounds present in higher concentrations (creosol, ethyl cyclohexanecarboxylate, and 4-ethyl acetophenone) allowed the six treatments to be grouped into three clusters. Cresol, a compound present in large amounts in all trials, is a compound commonly present in table olives produced by Seville style [42]. Low amounts of this compound may indicate the presence of alterative microorganisms [42]. The presence of creosol in fermented table olives is considered positive because this compound emits sweet, caramel, or vanilla-like odours [43]. The presence of high levels of cresol can neither be attributed to the type of nutrient used nor to the presence of the starter strain OM13, as it was detected in cluster 3 in both the inoculated experimental trials (NdB-3, NdB-4, and NdB-6) and the spontaneously fermented control (NdB-1). The odours associated with this molecule give the product its typical vinegary odour and bitter flavour [44]. Esters were the third group of VOCs with the highest concentration. Their presence is related to microbial activity. Esters are important molecules, as they are compounds that emit fruity and floral notes, so they have a positive impact on defining the odours and flavours of the final product [45]. The ester with the highest levels in NdB-2 (cluster 2) was ethyl cyclohexanecarboxylate, although it was also found in trials with nutrients C (NdB-5) and D (NdB-6). It is a chemical whose presence is related to the characteristic fruity odour prevalent in Spanish olives, due to its low perception threshold (0.001 μg/Kg) [46,47]. The only ketone detected was 4-Ethylacetophenone. Its presence in significant quantities in NdB-5 could be due to the nutrient C used (cluster 3). In previous studies on Nocellara del Belice table olives processed in the Sevillian [21] and Greek [31] styles, fermented with *L. pentosus* OM13, the amount determined was much lower (around 11–50 μg/kg). Its presence produces the emission of odours associated with hawthorn flowers (www.thegoodscentscompany.com, accessed on 13 March 2023). Presumably, nutrients—particularly nutrient D—increased acetic acid content, with the nutrient-free experimental trial (NdB-2) containing the lowest acetic acid content. When comparing acetic acid levels in trials where the nutrient was added with glucose (NdB-4, Ndb-5, and NdB-6) and the other trials, sugars did not affect acetic acid production. Among the alcohols, the largest amounts were detected in phenylethyl alcohol, which was present in all experimental productions except the spontaneously fermented control. Eshkol et al. [48] attributed the presence of this compound to the fermentation of *Saccharomyces cerevisiae* yeasts. Its presence is positive, as it imparts rose-like odours to table olives [49]. Benzaldehyde and alpha-cubenene are two other compounds found in higher quantities among the aldehydes and aromatic hydrocarbons, respectively. Benzaldehyde is a compound that is mainly synthesised by the enzymatic activity of LAB belonging to the genus *Lactobacillus*, while alpha-cubenene is terpene commonly associated to varietal aromas of olives [30,44]. The presence of different forms of alpha-amino nitrogen in three nutrients (B, C, and D) could influence the aromatic expression. With the same glucose, the use of inactivated yeasts enriched in mannoproteins (nutrient D) could have caused an increase in acetic acid, phenylethyl alcohol, phenylacetaldehyde, methyl hydrocinnamate, creosol, guaiacol, and phenol. On the other hand, in the trial with nutrient C (NdB-5), the highest levels were found for benzaldehyde, 2,5-dimethylbenzaldehyde, isophthaldehyde, styrene, butyrolactone, octyl acetate, phenylmethyl acetate, 4-ethylacetophenone, and phenol. In comparison to other experimental treatments, the addition of nutrient C has led to more aromaticity. In fact, all the compounds present in greater quantities are known for their ability to produce odours and flavours that range from floral to fruity, enriching the sensory profile of the trial NdB-5.

The results of the VOC study were validated by sensory analysis. In fact, NdB-5 achieved the highest score of overall acceptability—so much so that the clusters obtained by processing through AHC distinguished NdB-5 from the spontaneously fermented control (NdB-1) and all other treatments, which from the sensory aspect, were grouped into a single cluster and showed no difference between them. The high scores for crispness, green olive aroma, juicy, salt, and sweet in NdB-5 clearly indicate how *L. pentosus* OM13, in the presence of nutrient C, determined an improvement in organoleptic characteristics in Seville-style olives [44,50,51].

## 5. Conclusions

This study indicates the importance of nutritional adjuvants in significantly reducing the acidification process of olive brine during the 195 day fermentation, thereby improving the safety and sensory quality of the final product. This research has a significant potential for applicability in the industrial production of table olives. Indeed, rapid acidification results in a reduction in processing time, leading to lower production costs. Moreover, the rapid lowering of pH results in less product loss compared to table olives produced with traditional protocols. The different chemical composition of the nutrients clearly differentiated the experimental productions obtained on a chemical–physical, microbiological, and sensory level. Nutrient C, consisting of glucose, maltodextrins, and inactivated yeasts, improved the fermentation performance of *L. pentosus* OM13 compared to the other nutrients. In fact, fermented olives obtained with nutrient C showed high scores in the panel test. Further investigations will be carried out with a view toward an industrial-scale development of the optimized fermentation protocols.

## Figures and Tables

**Table 1:** Chemical composition of the four nutrients used in the production of ‘Nocellara del Belice’ Seville-style table olives.

Compounds (g/Kg)	Nutrient A	Nutrient B	Nutrient C	Nutrient D
Starch	550	0	0	0
Sugars (glucose)	200	400	400	400
Maltodextrine	0	500	500	500
Inactivated yeast	0	0	100	0
Inactivated yeast rich in amino acids	0	100	0	0
Inactivated yeast rich in mannoproteins	0	0	0	100
Salt (NaCl)	250	0	0	0

**Table 2:** pH values measured during fermentation of Nocellara del Belice table olives.

Fermentation Days	Trials						Statistical Significance
	NdB-1	NdB-2	NdB-3	NdB-4	NdB-5	NdB-6	
0	8.50 ± 0.02 ^a^	8.50 ± 0.01 ^a^	8.50 ± 0.02 ^a^	8.50 ± 0.03 ^a^	8.50 ± 0.02 ^a^	8.50 ± 0.03 ^a^	n.s.
3	7.14 ± 0.06 ^a^	6.47 ± 0.01 ^b^	6.05 ± 0.12 ^c^	6.03 ± 0.05 ^c^	6.01 ± 0.12 ^c^	6.04 ± 0.13 ^c^	***
6	6.77 ± 0.02 ^a^	6.02 ± 0.01 ^b^	5.71 ± 0.01 ^c^	5.68 ± 0.03 ^c^	5.70 ± 0.07 ^c^	5.66 ± 0.01 ^c^	***
9	6.68 ± 0.03 ^a^	5.64 ± 0.02 ^b^	5.44 ± 0.03 ^c^	5.04 ± 0.03 ^d^	5.04 ± 0.11 ^d^	4.96 ± 0.13 ^d^	***
15	6.29 ± 0.12 ^a^	5.11 ± 0.07 ^b^	5.00 ± 0.14 ^bc^	4.79 ± 0.14 ^d^	4.65 ± 0.09 ^d^	4.82 ± 0.04 ^cd^	***
35	5.82 ± 0.02 ^a^	4.98 ± 0.06 ^b^	4.87 ± 0.06 ^b^	4.58 ± 0.11 ^c^	4.48 ± 0.12 ^c^	4.51 ± 0.02 ^c^	***
65	5.49 ± 0.01 ^a^	4.68 ± 0.04 ^c^	4.79 ± 0.05 ^b^	4.51 ± 0.05 ^d^	4.39 ± 0.04 ^e^	4.48 ± 0.02 ^d^	***
143	5.28 ± 0.12 ^a^	4.73 ± 0.13 ^b^	4.65 ± 0.06 ^b^	4.47 ± 0.05 ^c^	4.37 ± 0.05 ^c^	4.48 ± 0.01 ^c^	***
195	4.93 ± 0.08 ^a^	4.54 ± 0.07 ^b^	4.54 ± 0.04 ^b^	4.47 ± 0.10 ^bc^	4.35 ± 0.05 ^c^	4.48 ± 0.09 ^bc^	***

Result indicate mean value ± standard deviation of three misurations. Abbreviations: n.s., not significant; NdB-1, spontaneous fermentation; NdB-2, *L. pentosus* OM13; NdB-3, *L. pentosus* OM13 + nutrient A; NdB-4, *L. pentosus* OM13 + nutrient B; NdB-5, *L. pentosus* OM13 + nutrient C; NdB-6, *L. pentosus* OM13 + nutrient D. Data in the same line by the same letter are not significantly different according to Tukey’s test. *p* value: ***, *p* < 0.001.

**Table 3:** Volatile organic compounds determined on the different experimental tests at the end of the production process (195 days).

Chemical Compounds	Trials						S.s ^1^
	NdB-1	Ndb-2	NdB-3	NdB-4	NdB-5	NdB-6	
Σ Acids	2793.08 ± 302.52 ^b^	1337.90 ± 160.82 ^c^	1455.16 ± 166.83 ^c^	2407.99 ± 184.84 ^b^	2082.21 ± 161.32 ^d^	3973.71 ± 599.66 ^a^	***
Acetic acid	1997.51 ± 249.70 ^b^	105.61 ± 9.37 ^d^	967.73 ± 107.37 ^c^	1891.65 ± 129.84 ^b^	1483.32 ± 97.81 ^b^	3190.57 ± 524.38 ^a^	***
Butanoic acid	63.44 ± 3.81 ^b^	443.75 ± 68.23 ^a^	66.71 ± 11.13 ^b^	54.89 ± 3.08 ^b^	61.28 ± 4.02 ^b^	56.15 ± 5.59 ^b^	***
Hexanoic acid	126.42 ± 0.91 ^c^	459.20 ± 65.82 ^a^	167.78 ± 13.09 ^c^	173.10 ± 21.52 ^c^	219.32 ± 19.09 ^b^	259.41 ± 27.46 ^b^	***
Pentanoic acid	0.00 ± 0.00 ^b^	196.17 ± 0.15 ^a^	0.00 ± 0.00 ^b^	0.00 ± 0.00 ^b^	0.00 ± 0.00 ^b^	0.00 ± 0.00 ^b^	***
Propionic acid	605.75 ± 48.10 ^a^	133.17 ± 17.25 ^e^	252.94 ± 35.23 ^d^	288.35 ± 30.41 ^d^	318.29 ± 40.40 ^c^	467.57 ± 42.23 ^b^	***
Σ Alcohols	2021.64 ± 164.88 ^b^	1428.48 ± 88.08 ^c^	1510.95 ± 118.47 ^c^	1879.68 ± 201.88 ^b^	360.31 ± 30.15 ^d^	2425.59 ± 192.48 ^a^	***
1-Butanol	0.00 ± 0.00 ^c^	56.00 ± 6.39 ^a^	0.00 ± 0.00 ^c^	0.00 ± 0.00 ^c^	34.19 ± 5.75 ^b^	0.00 ± 0.00 ^c^	***
3-Methyl-1-butanol	75.82 ± 11.94 ^a^	0.00 ± 0.00 ^c^	0.00 ± 0.00 ^c^	46.09 ± 8.55 ^b^	72.86 ± 3.65 ^a^	43.51 ± 1.31 ^b^	***
1-Hexanol	95.36 ± 17.71 ^ab^	94.44 ± 5.57 ^ab^	28.60 ± 4.87 ^c^	104.89 ± 12.25 ^a^	0.00 ± 0.00 ^d^	75.34 ± 12.42 ^ab^	***
1-Octanol	113.27 ± 9.56 ^a^	71.76 ± 10.90 ^b^	74.49 ± 2.42 ^b^	91.79 ± 7.25 ^b^	78.83 ± 6.95 ^b^	115.48 ± 5.66 ^a^	***
2-Butanol	684.42 ± 62.44 ^a^	146.18 ± 19.72 ^b^	0.00 ± 0.00 ^c^	0.00 ± 0.00 ^c^	0.00 ± 0.00 ^c^	0.00 ± 0.00 ^c^	***
3-Hexen-1-ol	408.24 ± 11.29 ^a^	288.72 ± 36.59 ^b^	306.72 ± 11.03 ^b^	337.28 ± 29.69 ^b^	125.15 ± 11.75 ^c^	315.00 ± 34.98 ^b^	***
Benzyl alcohol	644.53 ± 51.94 ^a^	307.50 ± 2.19 ^c^	403.18 ± 71.03 ^bc^	487.31 ± 54.81 ^b^	0.00 ± 0.00 ^d^	686.69 ± 53.32 ^a^	***
Phenylethyl alcohol	0.00 ± 0.00 ^e^	463.88 ± 6.72 ^d^	697.96 ± 29.11 ^c^	812.33 ± 89.34 ^b^	49.28 ± 2.05 ^e^	1189.57 ± 84.79 ^a^	***
Σ Aldehydes	422.53 ± 28.03 ^b^	346.26 ± 46.32 ^b^	243.96 ± 33.00 ^b^	301.59 ± 36.31 ^b^	2959.21 ± 372.18 ^a^	292.78 ± 28.99 ^b^	***
Benzaldehyde	232.00 ± 3.29 ^b^	132.91 ± 23.60 ^b^	134.78 ± 21.89 ^b^	150.83 ± 19.53 ^b^	2200.07 ± 307.83 ^a^	145.73 ± 15.71 ^b^	***
2,5-Dimethylbenzaldehyde	73.25 ± 8.85 ^b^	31.09 ± 1.69 ^cd^	41.93 ± 5.17 ^c^	22.15 ± 2.15 ^d^	119.39 ± 8.88 ^a^	0.00 ± 0.00 ^e^	***
Benzaldehyde-3-ethyl	52.92 ± 9.22 ^a^	27.96 ± 3.11 ^b^	30.23 ± 2.58 ^b^	26.73 ± 3.95 ^b^	18.88 ± 0.64 ^b^	19.50 ± 2.44 ^b^	**
Isophthaldehyde	0.00 ± 0.00 ^b^	28.20 ± 0.65 ^b^	37.03 ± 3.36 ^b^	23.52 ± 0.77 ^b^	265.11 ± 36.69 ^a^	19.78 ± 1.52 ^b^	***
Nonal	0.00 ± 0.00 ^d^	84.89 ± 15.27 ^a^	0.00 ± 0.00 ^d^	25.33 ± 2.03 ^c^	0.00 ± 0.00 ^d^	41.69 ± 1.30 ^b^	***
Octanal	50.45 ± 4.01 ^b^	0.00 ± 0.00 ^c^	0.00 ± 0.00 ^c^	0.00 ± 0.00 ^c^	335.42 ± 16.11 ^a^	0.00 ± 0.00 ^c^	***
Phenylacetaldehyde	13.92 ± 2.67 ^d^	41.22 ± 2.01 ^c^	0.00 ± 0.00 ^e^	53.03 ± 7.88 ^b^	20.36 ± 2.02 ^d^	66.09 ± 8.03 ^a^	***
Σ Aromatic hydrocarbons	173.47 ± 13.22 ^a^	136.45 ± 13.04 ^b^	38.11 ± 3.54 ^d^	105.19 ± 12.80 ^c^	141.73 ± 12.54 ^ab^	155.92 ± 10.55 ^ab^	***
Alpha-cubebene	125.17 ± 8.62 ^a^	71.34 ± 6.82 ^b^	38.11 ± 3.54 ^c^	66.68 ± 7.20 ^b^	40.95 ± 2.48 ^c^	70.50 ± 3.86 ^b^	***
Styrene	48.30 ± 4.60 ^d^	65.11 ± 6.22 ^c^	0.00 ± 0.00 ^e^	38.50 ± 5.60 ^d^	100.78 ± 10.06 ^a^	85.42 ± 6.69 ^b^	***
Σ Esters	1233.08 ± 89.05 ^c^	6389.72 ± 453.45 ^a^	296.99 ± 37.22 ^d^	362.92 ± 54.56 ^d^	2209.58 ± 252.43 ^b^	472.18 ± 53.72 ^d^	***
Butyrolactone	29.23 ± 3.46 ^b^	30.38 ± 3.01 ^b^	37.72 ± 4.06 ^b^	36.78 ± 3.80 ^b^	451.76 ± 50.78 ^a^	59.12 ± 6.54 ^b^	***
cis-3-Hexenylacetate	106.30 ± 26.90 ^abc^	133.41 ± 13.45 ^ab^	91.06 ± 6.68 ^bc^	158.16 ± 32.39 ^a^	70.69 ± 4.93 ^c^	160.68 ± 12.41 ^a^	***
Ethyl cyclohexanecaboxlate	0.00 ± 0.00 ^b^	3036.13 ± 289.05 ^a^	0.00 ± 0.00 ^b^	0.00 ± 0.00 ^b^	30.04 ± 7.04 ^b^	19.50 ± 1.54 ^b^	***
Ethyl dihydrocinnamate	989.23 ± 49.23 ^a^	0.00 ± 0.00 ^c^	0.00 ± 0.00 ^c^	0.00 ± 0.00 ^c^	860.63 ± 95.32 ^b^	0.00 ± 0.00 ^c^	***
Ethyl hexanoate	0.00 ± 0.00 ^b^	1076.68 ± 65.44 ^a^	0.00 ± 0.00 ^b^	0.00 ± 0.00 ^b^	22.47 ± 2.95 ^b^	0.00 ± 0.00 ^b^	***
Ethyl pentanoate	0.00 ± 0.00 ^c^	395.66 ± 34.21 ^a^	0.00 ± 0.00 ^c^	0.00 ± 0.00 ^c^	39.70 ± 3.62 ^b^	0.00 ± 0.00 ^c^	***
Methyl cyclohexanoate	0.00 ± 0.00 ^b^	1226.60 ± 10.51 ^a^	0.00 ± 0.00 ^b^	0.00 ± 0.00 ^b^	0.00 ± 0.00 ^b^	0.00 ± 0.00 ^b^	***
Methyl hexanoate	0.00 ± 0.00 ^b^	346.67 ± 26.92 ^a^	0.00 ± 0.00 ^b^	0.00 ± 0.00 ^b^	0.00 ± 0.00 ^b^	0.00 ± 0.00 ^b^	***
Methyl hydrocinnamate	22.69 ± 1.19 ^b^	14.79 ± 1.54 ^c^	15.12 ± 1.52 ^c^	0.00 ± 0.00 ^d^	0.00 ± 0.00 ^d^	41.82 ± 2.59 ^a^	***
Octyl acetate	31.85 ± 5.03 ^c^	16.24 ± 1.44 ^c^	18.80 ± 1.46 ^c^	30.74 ± 3.35 ^c^	191.47 ± 23.87 ^a^	108.99 ± 15.34 ^b^	***
Phenylmethyl acetate	53.77 ± 3.23 ^bc^	0.00 ± 0.00 c	92.74 ± 19.24 ^b^	77.72 ± 10.13 ^b^	503.10 ± 62.05 ^a^	82.07 ± 15.31 ^b^	***
Propyl butyrate	0.00 ± 0.00 ^d^	113.16 ± 7.90 ^a^	41.56 ± 4.26 ^c^	59.53 ± 4.91 ^b^	39.73 ± 1.87 ^c^	0.00 ± 0.00 ^d^	***
Σ Ketones	202.83 ± 39.42 ^b^	180.45 ± 11.68 ^b^	142.60 ± 11.33 ^b^	138.91 ± 21.79 ^b^	3782.91 ± 421.32 ^a^	160.74 ± 10.46 ^b^	***
4-Ethylacetophenone	202.83 ± 39.42 ^b^	180.45 ± 11.68 ^b^	142.60 ± 11.33 ^b^	138.91 ± 21.79 ^b^	3782.91 ± 421.32 ^a^	160.74 ± 10.46 ^b^	***
Σ Phenols	4430.94 ± 300.82 ^b^	3052.54 ± 181.17 ^d^	3191.54 ± 162.06 ^cd^	3703.29 ± 342.62 ^c^	264.68 ± 38.61 ^e^	7300.78 ± 306.86 ^a^	***
Creosol	4217.22 ± 278.65 ^b^	2302.65 ± 107.69 ^d^	3000.63 ± 138.77 ^c^	3106.17 ± 286.19 ^c^	23.16 ± 1.23 ^e^	6176.69 ± 230.55 ^a^	***
Guaiacol	80.82 ± 14.33 ^d^	272.30 ± 21.75 ^c^	99.11 ± 16.68 ^d^	486.15 ± 45.37 ^b^	62.36 ± 9.15 ^d^	900.64 ± 40.43 ^a^	***
Phenol	52.68 ± 4.12 ^b^	31.73 ± 4.32 ^b^	39.48 ± 1.51 ^b^	56.07 ± 7.04 ^b^	133.57 ± 24.88 ^a^	111.63 ± 22.60 ^a^	***
p-Creosol	80.22 ± 3.72 ^bc^	445.86 ± 47.40 ^a^	52.33 ± 5.11 ^c^	54.90 ± 4.02 ^c^	45.59 ± 3.35 ^c^	111.82 ± 13.28 ^b^	***

^1^ Statistical significance. According to Tukey’s test, data within a line followed by the same letter are not significantly different. *p* value: ***, *p* < 0.001; **, *p* < 0.01. Abbreviations: NdB-1, spontaneous fermentation; NdB-2, *L. pentosus* OM13; NdB-3, *L. pentosus* OM13 + nutrient A; NdB-4, *L. pentosus* OM13 + nutrient B; NdB-5, *L. pentosus* OM13 + nutrient C; NdB-6, *L. pentosus* OM13 + nutrient D. ** explanation.

**Table 4:** Evaluation of the sensory attributes of the experimental table olives.

Attributes	Trials ^1^							
	NdB-1	NdB-2	NdB-3	NdB-4	NdB-5	NdB-6	SEM ^2^	S.s ^3^
Acid	2.35 ^b^	4.48 ^a^	3.89 ^a^	4.15 ^a^	2.37 ^b^	4.03 ^a^	0.22	***
Astringent	1.48 ^b^	1.95 ^a^	1.81 ^ab^	1.47 ^b^	1.58 ^ab^	1.55 ^ab^	0.05	**
Bitter	2.82 ^a^	3.42 ^a^	3.15 ^a^	3.19 ^a^	2.62 ^a^	2.98 ^a^	0.07	n.s.^4^
Brightness	4.20 ^a^	4.55 ^a^	4.03 ^a^	4.11 ^a^	3.92 ^a^	4.19 ^a^	0.05	n.s.^4^
Crispness	4.23 ^b^	7.18 ^a^	6.82 ^a^	6.95 ^a^	7.00 ^a^	6.88 ^a^	0.26	*
Green colour intensity	6.27 ^a^	5.25 ^a^	5.33 ^a^	5.35 ^a^	6.17 ^a^	5.86 ^a^	0.11	n.s.^4^
Green olive aroma	1.83 ^b^	5.60 ^a^	5.15 ^a^	5.37 ^a^	6.13 ^a^	5.42 ^a^	0.36	*
Juicy	3.38 ^c^	4.38 ^bc^	5.33 ^ab^	5.49 ^ab^	6.18 ^a^	5.17 ^ab^	0.28	***
Off-flavours	4.82 ^a^	2.19 ^b^	1.51 ^b^	1.38 ^b^	1.13 ^b^	1.27 ^b^	0.33	***
Off-odours	3.27 ^a^	1.52 ^b^	1.44 ^b^	1.39 ^b^	1.17 ^b^	1.33 ^b^	0.18	***
Salt	5.97 ^a^	5.03 ^b^	5.81 ^a^	4.98 ^b^	6.13 ^a^	6.46 ^a^	0.14	***
Sweet	1.33 ^d^	2.68 ^bc^	2.22 ^c^	2.06 ^c^	4.32 ^a^	2.87 ^ab^	0.16	***
Overall acceptability	3.55 ^c^	5.55 ^bc^	5.39 ^bc^	6.48 ^ab^	7.80 ^a^	6.07 ^ab^	0.30	**

Data in the same line followed by the same letter are not significantly different according to Tukey’s test. *p* value: ***, *p* < 0.001; **, *p* < 0.01; *, *p* < 0.05. ^1^ Trial codes: NdB-1, spontaneous fermentation; NdB-2, *L. pentosus* OM13; NdB-3, *L. pentosus* OM13 + nutrient A; NdB-4, *L. pentosus* OM13 + nutrient B; NdB-5, *L. pentosus* OM13 + nutrient C; NdB-6, *L. pentosus* OM13 + nutrient D. ^2^ Standard error of the mean. ^3^ Statistical significance. ^4^ Not significant.

## Data Availability

All data included in this study are available upon request by contacting the corresponding author.
